# Genome-resolved metagenomics of eukaryotic populations during early colonization of premature infants and in hospital rooms

**DOI:** 10.1186/s40168-019-0638-1

**Published:** 2019-02-15

**Authors:** Matthew R. Olm, Patrick T. West, Brandon Brooks, Brian A. Firek, Robyn Baker, Michael J. Morowitz, Jillian F. Banfield

**Affiliations:** 1 Department of Plant and Microbial Biology, University of California, Berkeley, CA USA; 2 Department of Surgery, University of Pittsburgh School of Medicine, Pittsburgh, PA USA; 3 Division of Newborn Medicine, Magee-Womens Hospital of UPMC, Pittsburgh, PA USA; 4 Department of Earth and Planetary Science, University of California, Berkeley, CA USA; 5 Department of Environmental Science, Policy, and Management, University of California, Berkeley, CA USA; 6 Earth Sciences Division, Lawrence Berkeley National Laboratory, Berkeley, CA USA; 7 Chan Zuckerberg Biohub, San Francisco, CA USA; 8 Present address: Kaleido Biosciences, Bedford, MA USA

**Keywords:** Eukaryotes, Metagenomics, Genome-resolved metagenomics, Hospital microbiome, Neonatal intensive care unit, Premature infants

## Abstract

**Background:**

Fungal infections are a significant cause of mortality and morbidity in hospitalized preterm infants, yet little is known about eukaryotic colonization of infants and of the neonatal intensive care unit as a possible source of colonizing strains. This is partly because microbiome studies often utilize bacterial 16S rRNA marker gene sequencing, a technique that is blind to eukaryotic organisms. Knowledge gaps exist regarding the phylogeny and microdiversity of eukaryotes that colonize hospitalized infants, as well as potential reservoirs of eukaryotes in the hospital room built environment.

**Results:**

Genome-resolved analysis of 1174 time-series fecal metagenomes from 161 premature infants revealed fungal colonization of 10 infants. Relative abundance levels reached as high as 97% and were significantly higher in the first weeks of life (*p* = 0.004). When fungal colonization occurred, multiple species were present more often than expected by random chance (*p* = 0.008). Twenty-four metagenomic samples were analyzed from hospital rooms of six different infants. Compared to floor and surface samples, hospital sinks hosted diverse and highly variable communities containing genomically novel species, including from *Diptera* (fly) and *Rhabditida* (worm) for which genomes were assembled. With the exception of *Diptera* and two other organisms, zygosity of the newly assembled diploid eukaryote genomes was low. Interestingly, *Malassezia* and *Candida* species were present in both room and infant gut samples.

**Conclusions:**

Increased levels of fungal co-colonization may reflect synergistic interactions or differences in infant susceptibility to fungal colonization. Discovery of eukaryotic organisms that have not been sequenced previously highlights the benefit of genome-resolved analyses, and low zygosity of assembled genomes could reflect inbreeding or strong selection imposed by room conditions.

**Electronic supplementary material:**

The online version of this article (10.1186/s40168-019-0638-1) contains supplementary material, which is available to authorized users.

## Background

Eukaryotes are common members of the human microbiome [1–3]. The colonization density and diversity of eukaryotes are lower than their bacterial counterparts [1, 4, 5], but they can have substantial health consequences. The yeast *Saccharomyces boulardii* can significantly reduce rates of antibiotic-associated diarrhea [6], protozoa limit *Salmonella* populations through predation [7], and high abundances of *Candida* and *Rhodotorula* are associated with asthma development in neonates [8]. Fungal disease is most prevalent in immunocompromised patients, including premature infants [9, 10], although their incidence has declined in recent decades [11].

While infant fungal disease is an active area of study, little is known about asymptomatic colonization of premature infants by fungi or other eukaryotes. Studies have reported 0%, 26%, 50%, and 63% of premature infants being colonized by fungi [2, 12–14], with variation in methodological sensitivity probably at the heart of these differences. Methods used to analyze the mycobiome, including culturing, DGGE, and ITS sequencing, identify the fungal fraction of the microbial community separate from the community at large. This has left basic knowledge gaps about the relative abundance of fungi in early life, an important point as fungi-infant interactions in early life are known to affect allergy development [8, 15, 16]. In fact, recent review articles have referred to eukaryotes as a “Missing Link in Gut Microbiome Studies” [17], and stated that “Studies addressing how the infant mycobiome develops and shapes the host immune system will be required for a more comprehensive understanding of the early-life microbiome.” [3]. Particular highlighted knowledge gaps relate to the ecological roles, growth dynamics, and source of eukaryotes in the human and hospital microbiomes [17, 18].

The hospital is a known source for bacterial infant colonists [19]. The built environment has been implicated in fungal outbreaks [20–23], yet the eukaryotic built environment microbiome remains understudied. This is because the vast majority of high-throughput studies of the hospital microbiome and the human gut microbiome use bacteria-specific 16S rRNA marker gene sequencing, and thus are blind to eukaryotes. Of five recent studies of the hospital microbiome, only one included primers to target the internal transcribed spacer (ITS) sequences to detect eukaryotes [24–28]. It remains to be seen if eukaryotes in the room have the genetic potential to colonize infants, and if so, where in the room these eukaryotes are located.

An alternative approach to microbiome characterization involves shotgun metagenomics. In this method, all DNA from a sample is sequenced regardless of its organismal source or genetic context. In some studies, mapping of the sequencing reads to reference genomes has enabled identification of pathogens [29]. However, the reads can be assembled, and new methods aid in reconstructing eukaryotic genomes from these datasets [30], enabling understanding of these organisms in the context of their entire communities, which also include bacteria, archaea, bacteriophage, viruses, and plasmids. Relative to amplicon sequencing, genome assembly has several distinct advantages for understanding communities that contain eukaryotes. First, genomes provide information about in situ ploidy (number of distinct chromosome sets per cell), heterozygosity (here used to refer to the fraction of alleles in a diploid genome that have two versus one abundant sequence types), and extent of population microdiversity (here used to refer to additional sequence types that constitute low-abundance alleles). Second, strain-tracking can be performed using high-resolution genomic comparisons. Last, newly assembled eukaryotic sequences expand the diversity of genomically defined eukaryotes in public databases, enabling comparative and evolutionary studies.

Here, we used genome-resolved metagenomics to study eukaryote-containing microbiomes of premature infants and their NICU environment. We evaluated the incidence of eukaryotes in room and infant samples and investigated the time period during which infant microbiomes contained eukaryotes. Genomes were assembled for 14 eukaryotic populations, and their ploidy, zygosity, and population microdiversity defined. The same species of eukaryotes were found in infant microbiome and the NICU environment, and a subset of other microbial eukaryotes in NICU rooms was classified as types that can cause nosocomial infections.

## Results

### Recovery of novel eukaryotic genomes from metagenomes

In this study, we analyzed 1174 fecal metagenomes and 24 metagenomes from the NICU environment, totaling 5.31 Tb of DNA sequence **(**Additional file 1: Table S1**)**. Fecal samples were collected from 161 premature infants primarily during the first 30 days of life (DOL) (full range of DOL 5–121; median 18), with an average of 7 samples per infant. NICU samples were taken from six patient rooms within the hospital housing the infants (Magee-Womens Hospital of UPMC, Pittsburgh, PA, USA). Three NICU locations were sampled in each room: swabs from frequently touched surfaces, wipes from other surfaces, and swabs from sinks [19]. Eukaryotic genomes were assembled from all samples using a EukRep-based pipeline ([30]; see the “Methods” section for details). The bacterial component of some of the datasets was analyzed previously (see the “Methods” section).

**Fig. 1:**
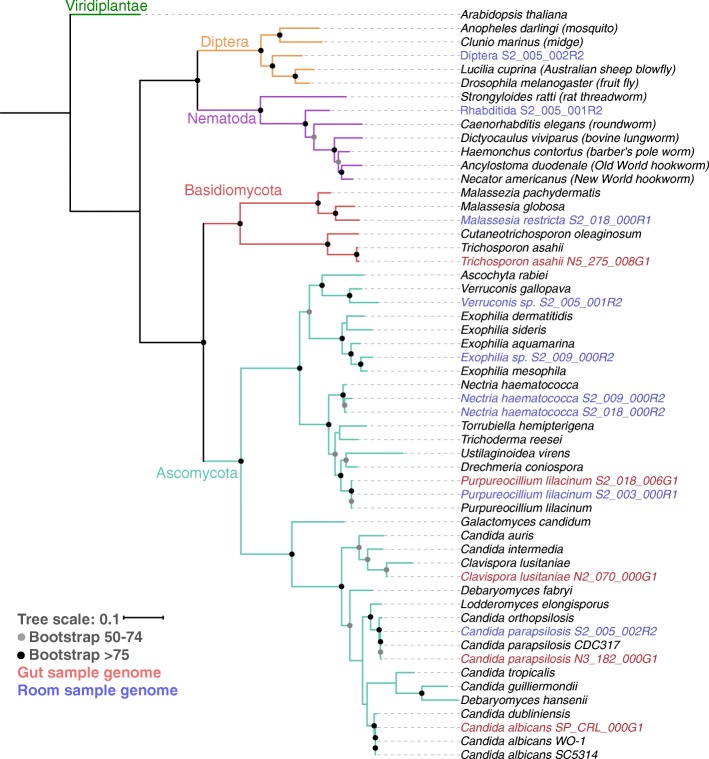
Phylogenetic tree of recovered eukaryote genomes. Genomes from infant-derived fecal samples (red) and NICU samples (blue) were classified using a phylogenetic tree based on the concatenation of the sequences of 16 ribosomal proteins (see the “Methods” section). Branches with greater than 50% bootstrap support are labeled with their bootstrap support range. Reference ribosomal protein sequences were obtained from NCBI [30] and the Candida Genome Database [30]

Fourteen novel eukaryotic genomes were recovered in total, with a median estimated completeness of 91% (Table 1). Detailed genome assembly information is available in Additional file 2: Table S2. Genomes were assembled from organisms of a wide phylogenetic breadth, and four are the first genome sequences for their species (Fig. 1). Twelve of the genomes are classified as fungal and are described in more detail below. The two other genomes (both recovered from hospital sink samples) represent the first genomes of their phylogenetic families. *Diptera* S2_005_002R2 is within the phylogenetic clade of Diptera (true flies) and is equally related to *Drosophila melanogaster* (fruit fly) and *Lucila cuprina* (Australian sheep blowfly). *Rhabditida* S2_005_001R2 is within the family Rhabditida (nematode) and is related to both pathogenic and non-pathogenic roundworms. In both cases, BLAST searches of the rpS3 protein sequence against NCBI revealed no significant hits, and furthermore, comparing the mitochondrial cytochrome c oxidase subunit I gene and protein against the Barcode Of Life Database (BOLD) [31] and NCBI revealed no hits with high identity. Thus, we are unable to tie our genomes to any morphologically described species.

**Table 1:** Description of de novo assembled eukaryotic genomes

Source	Genome	Completeness (%)	Length (bp)	N50 (bp)	Coverage
Infant gut	*Purpureocillium lilacinum* S2_018_006G1	98.4	35,688,710	422,361	20×
Infant gut	*Clavispora lusitania*e N2_070_000G1	95.8	11,907,650	89,311	18×
Infant gut	*Candida parapsilosis* N3_182_000G1	96.7	12,563,647	65,710	182×
Infant gut	*Trichosporon asahii* N5_275_008G1	90.1	23,419,590	32,912	13×
Infant gut	*Candida albicans* SP_CRL_000G1	91.1	12,561,678	22,840	30×
NICU room	*Purpureocillium lilacinum* S2_003_000R1	98.4	35,724,498	520,486	67×
NICU room	*Malassezia restricta* S2_018_000R1	72.6	6,457,898	4912	18×
NICU sink	*Nectria haematococca* S2_018_000R2	96.7	44,952,822	24,418	10×
NICU sink	*Candida parapsilosis* S2_005_002R2	92.8	11,573,959	14,507	9×
NICU sink	*Rhabditida* S2_005_001R2	74.9	50,505,025	8214	8×
NICU sink	*Nectria haematococca* S2_009_000R2	73.6	31,143,909	8000	7×
NICU sink	*Exophiala* sp. S2_009_000R2	75.9	24,670,482	7386	7×
NICU sink	*Diptera* S2_005_002R2	52.5	43,769,201	6834	10×
NICU sink	*Verruconis* sp. S2_005_001R2	52.8	15,639,153	5112	6×

### Fungal contaminants in extraction controls

Four negative extraction controls were subjected to metagenomic sequencing to detect sequences resulting from reagent contamination. One of the four extraction controls harbored *Purpureocillium lilacinum* DNA, with > 50% of sample reads mapping to the genome and with a breadth of coverage (percentage of the genome covered by at least one read) of 87% (Additional file 3: Figure S1A). The average nucleotide identity (ANI) was calculated between *P. lilacinum* reads in the extraction control, *P. lilacinum* genomes assembled in the study, and all previously sequenced *P. lilacinum* genomes in NCBI (Additional file 3: Figure S1B). *P. lilacinum* reads from the extraction control were extremely similar to genomes assembled from the NICU and infant gut, and divergent from previously sequenced genomes (Additional file 3: Figure S1B). Thus, *P. lilacinum* genomes assembled from room and gut samples are probably due to reagent contamination and not actually present in the environment.

Reads from three of the four extraction controls mapped to *Malassezia restricta* S2_018_000R1, all at low abundance (< 3% of reads with a genome breadth of coverage of 1.3–14.2% using reads from the four samples) (Additional file 3: Figure S1C). It was not possible to calculate the ANI between the genomes in samples and controls due to the low sequencing coverage of *Malassezia restricta* S2_018_000R1 in the extraction controls. *Malassezia* is a near-ubiquitous skin-associated fungus [32]. Based on the depth of coverage (2.37×), the genome had a very low breadth of coverage (88% expected vs. 13% actual) (Additional file 4: Figure S9), indicating that the genome sampled from the hospital surface is different to that of the *Malassezia* that contaminated the reagents. For this reason, the *Malassezia* in infant and room samples were not excluded from further analysis.

### Fungal microbiome of the premature infant gut

**Fig. 2:**
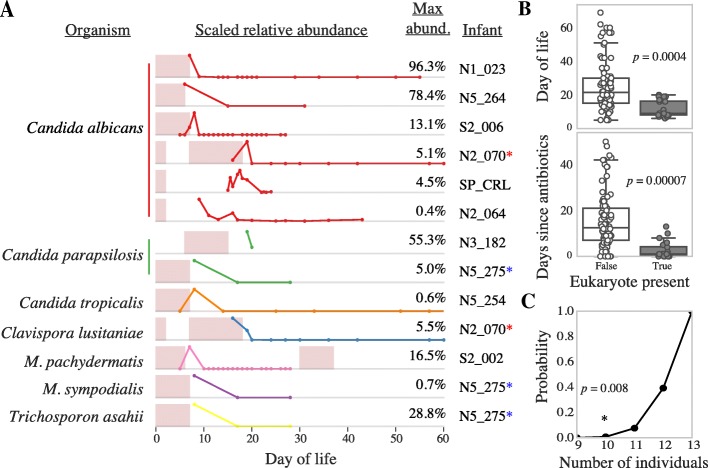
Abundance of eukaryotes colonizing infants. **a** The scaled relative abundance of each eukaryote colonizing an infant. Numbers on the right indicate the maximum relative abundance of the organism in that infant, and gray dividing lines indicate 0% relative abundance. Dots on the line-plots indicate days of life on which fecal samples were collected and sequenced. Infants colonized by multiple eukaryotes are marked with a colored asterisk. Pink bars indicate periods of antibiotic administration. **b** Metadata significantly associated with eukaryote abundance. The distribution of values for all samples in which eukaryotes are not present (left; white box plot) compared to values of samples in which eukaryotes are present (right; gray box plot). The *p* values were calculated using the Wilcoxon rank-sum test with Benjamini-Hochberg multiple testing *p* value correction. *P. lilacinum* was excluded from statistical tests due to its likely contaminant status. **c** Fungi are distributed among fewer individuals than expected by random chance. A permutation test was performed to determine the probability of observing 10 or less unique individuals colonized by 13 fungi from a population of 161 individuals. The number of unique individuals colonized is shown on the *x*-axis, and the empirical *p* value based on 100,000 trials is shown on the *y*-axis. An asterisk marks the true number of unique infants colonized in this study (10) and the associated *p* value

Excluding *P. lilacinum*, fungi were detected in 10 of the 161 premature infants profiled in this study (6%) (Fig. 2a; Additional file 5: Table S3). The limit of detection for eukaryotic organisms was calculated as 0.05% of the total community (Additional file 6: Figure S2) (see the “Methods” section for details). Eukaryotes were detected significantly more often early in life, and significantly more often when antibiotics were recently administered (Fig. 2b). Antibiotics were given significantly more often early in life (*p* = 5.3E−8; Wilcoxon rank-sum test), making it difficult to determine which of these two variables is driving the association.

Fungal colonization was not significantly associated with gestational age, twin status, birth weight, mode of delivery, or other clinical metadata. (Additional file 7: Tables S4, Additional file 8: Table S5)**.** Further, fungal colonization was not associated with bacterial community composition. *P. lilacinum*, presumed to be a metagenomic contaminant (Additional file 3: Figure S1), decreases in abundance as infants age (Additional file 9: Figure S8), probably because increased bacterial biomass in later collected samples overwhelms the contaminant DNA, as shown previously [33]. Given this, we infer that the decrease in relative abundance of fungi present in the microbiomes of later-collected samples is due to bacterial growth.

All seven species detected colonizing the premature infants have been previously implicated as agents of nosocomial infection (Table 2), yet no infants colonized by eukaryotes in this study received antifungals or showed any symptoms consistent with acute fungal infection. However, asymptomatic colonization has been shown to be a risk factor for future fungemia [34]. Seven different eukaryotic species were detected in at least one infant, with only *Candida albicans* and *Candida parapsilosis* colonizing more than one infant (Fig. 2a). Infant N2_070 was colonized by two fungi, and infant N5_275 was colonized by three. A permutation test was performed to determine if fungi were unevenly distributed among the infants of this study (i.e., if having one fungi predisposes colonization by another). The probability of observing 13 fungi colonize ≤ 10 unique individuals from a total population of 161 individuals was determined (Fig. 2c), with a resulting *p* value of 0.008. Thus, in this study, multiple fungi colonized the same infant more often than expected random chance.

**Table 2:** Description of detected fungal taxa

Taxa	Common habitats	Pathogenicity	Number of infants	Locations In NICU	Refs
*Candida albicans*	Warm blooded animals	Common nosocomial pathogen	6	Undetected	[1]
*Candida parapsilosis*	Warm blooded animals	Common nosocomial pathogen (especially neonates)	2	Sink	[82]
*Candida tropicalis*	Warm blooded animals	Common nosocomial pathogen	1	Undetected	[83]
*Nectria haematococca*	Soil, rhizosphere	Pathogen of immunocompromised patients	0	Sink	[84]
*Malassezia sympodialis*	Human skin	Opportunistic pathogen	1	Undetected	[85]
*Malassezia globosa*	Human skin	Common commensal; implicated in dandruff	0	Surfaces	[86]
*Malassezia pachydermatis*	Skin of mammals	Opportunistic pathogen	1	Undetected	[87]
*Trichosporon asahii*	Soil, human skin and GI tract	Rare opportunistic pathogen	1	Undetected	[88]
Verruconis	Soil, decaying vegetation	Verruconis includes black yeasts; human pathogens	0	Sink	[89]
Exophiala	Sinks, drain pipes, swimming pools	Exophiala contains pathogens of vertebrates	0	Sink	[90]

### Fungal microbiome of the neonatal intensive care unit

**Fig. 3:**
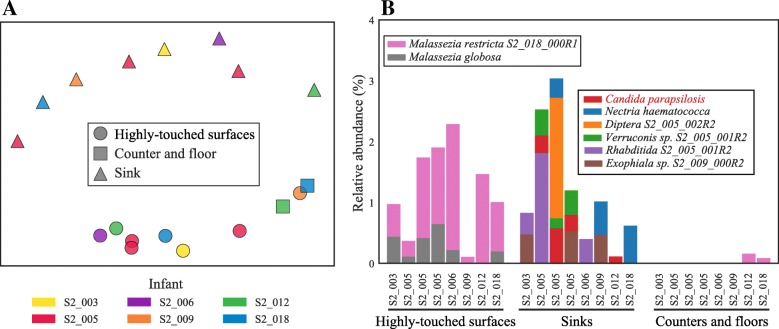
Eukaryotic microbiome of the neonatal intensive care unit (NICU). **a** Multidimensional scaling (MDS) of the Bray-Curtis dissimilarity between all NICU samples. Samples cluster by environment type rather than the room or occupant. The stress of the MDS was calculated to be 0.23. **b** Compositional profile of eukaryotic organisms detected in the NICU. Each colored box represents the percentage of reads mapping to an organism’s genome, and the stacked boxes for each sample show the fraction of reads in that dataset accounted for by different eukaryotic genomes in each sample

Eukaryotic organisms were detected in 18 of the 24 metagenomes of the NICU room environment (Fig. 3). Eukaryotic DNA made up an average of 1.23%, 1.22%, and 0.03% of the communities in highly-touched surfaces, sinks, and counters and floors, respectively. In order to compare the influence of room occupants and sampling location on the room mycobiome, we performed a multidimensional scaling (MDS) analysis (Fig. 3a**)**. Communities were differentiated based on sampling location rather than infant room.

The mycobiome of the NICU surfaces is dominated by species of *Malassezia* (Fig. 3b). The eukaryotic organisms found in NICU sinks are distinct from, and more diverse than, those found on surfaces. Sink communities contained *Necteria haematococca*, *Candida parapsilosis*, *Exophiala*, and *Verruconis*, all of which were detected in multiple rooms and samples. Additionally, sinks in three separate NICU rooms contain DNA from *Rhabditidia* S2_005_000R1 (a novel nematode; see the previous section for details). *Diptera* S2_005_002R2 (fly) also makes up about 2% of the entire community for a single time-point in the sink in infant S2_005’s room (Fig. 3b). No macroscopic organisms were noted during the sample collection process. It remains to be seen whether these organisms contribute to the dispersal of organisms throughout the NICU or affect the communities themselves.

*Candida parapsilosis* was detected in both the NICU and in a premature infant, as were organisms of the genus *Malassezia*. To contextualize the similarity between *C. parapsilosis* strains in both communities, genomes assembled from both the infant and room environments were compared to all available reference genomes and each other using dRep [35]. *C. parapsilosis* genomes from the NICU sink of infant S2_005 and gut of infant N3_182 were more similar to reference genomes than each other (Additional file 10: Figure S3), and thus do not represent direct strain transfer events.

### Sequence analysis of new genomes

De novo assembly of eukaryotic genomes from metagenomes allows not only for the detailed genomic comparison and detection of novel organisms, but also for the determination of ploidy, aneuploidy (abnormal number of chromosomes in a cell), heterozygosity, and population microdiversity of organisms in vivo. Changes in ploidy and aneuploidy have been observed in many eukaryotes, especially yeasts [36, 37], and are thought to be a strategy for relatively quick adaptation to shifts in environmental conditions. To determine the ploidy of genomes reconstructed in this study (Table 1), we examined the read count for each allele at a given variant site. For a diploid genome, alleles are expected to have a read count of 50%; for a triploid genome, alleles are expected to have a read count of either 33% or 67%. At low coverage, determining allele frequency with read mapping has more stochasticity relative to high coverage. Simulated reads for haploid, diploid, and triploid genomes at 10× and 100× coverage suggest it is possible to determine ploidy in even our low coverage genomes (Additional file 11: Figure S4). Based upon this analysis, all but one of our reconstructed genomes are diploid (Additional file 12: Figure S5). *C. lusitaniae* is likely haploid. Similarly, aneuploidy can be detected by searching for regions where allele frequencies and/or read coverage differs from the rest of the genome. Given the possibility of a parasexual cycle in *C. albicans* [38], detecting aneuploidy was of particular interest. We searched for evidence of aneuploidy using both our reconstructed genomes and reference genomes, but did not see evidence for aneuploidy in any of our genomes using either method (Additional file 13: Figure S6, Additional file 14: Figure S7).

**Fig. 4:**
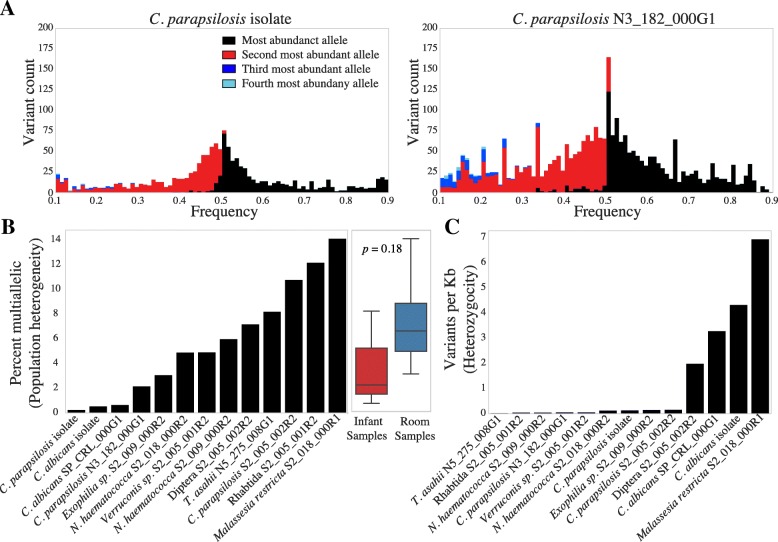
Ploidy, zygosity, and microdiversity of recovered eukaryotic genomes. **a** Histogram of the frequencies of the four most abundant variants at each variant site in an isolate genome of *C. parapsilosis* and in a genome of *C. parapsilosis* recovered in this study. Black, red, dark blue, and light blue bars indicate the abundances of the most abundant, second, third, and fourth most abundant variant, respectively. **b** For each genome, black bars indicate the percentage of variant sites that are multiallelic (contain more variants at a site than would be expected based upon ploidy alone). Haplotypes with more than two alleles are also considered to be multiallelic. A box plot compares the values from genomes originating from infant guts vs. the NICU room. **c** For each genome, black bars indicate the number of heterozygous variants per kb across the entire assembled genome

For diploid genomes reconstructed from metagenomes, the sequences for each chromosome are a composite of sequences from the two alleles. Population microdiversity can be detected based on read counts that exceed the expected ratio of 50%. Measuring population microdiversity in this way can be confounded by sequencing error and stochastic read coverage variation (Additional file 11: Figure S4). Genomic datasets for isolates are not expected to have population microdiversity but will display sequencing error and stochastic read coverage variation. Consequently, we could separate sequencing noise from true population microdiversity by comparing the patterns we observed in our population genomic data to microdiversity found in isolate genomic datasets [39]. For *C. parapsilosis* N3_182_000G1, the peak of allele frequencies is wider than that of the sequenced *Candida parapsilosis* isolate (Fig. 4a), suggesting considerable population microdiversity. The *P. lilacinum* contaminant also displayed substantial microdiversity (Additional file 15: Figure S10). To avoid the stochasticity introduced by low sequencing coverage (Additional file 11: Figure S4), only genomes with over 50× sequencing coverage were analyzed for population microdiversity in this way.

Another method of measuring population microdiversity involves determining the number of multiallelic sites (sites with more than two sequence variants). Tests with simulated reads were performed to confirm that non-specific mapping of reads from unrelated species does not bias results (see the “Methods” section). All of our genomes have more multiallelic sites than isolate-sequenced genomes (Fig. 4b), suggesting that all of our genomes have appreciable population microdiversity. Further, genomes from the room had higher microdiversity than those from the gut, although this comparison is not statistically significant (*p* = 0.09).

Finally, overall heterozygosity for each genome was measured by calculating the number of heterozygous SNPs per kilo-base pair (Fig. 4c). A wide range of heterozygosity was observed within genomes. For most organisms, there was low heterozygosity, and for *C. albicans* and *C. parapsilosis*, comparable to that of reference isolates. *Malassezia restricta* S2_018_000R1 has both a particularly high rate of SNPs per kilo-base pair and high population microdiversity.

## Discussion

### Eukaryotic genome recovery from metagenomes augments information from isolate studies

In contrast with prior studies that have investigated microbial eukaryote genomes via sequencing of isolates, we employed a whole-community sequencing approach and could detect population microdiversity in both NICU and infant-derived samples. *Malassezia* on NICU surfaces has particularly high population microdiversity. Given that *Malassezia* are skin-associated fungi [32], their high population microdiversity may be the consequence of the accumulation of numerous strains throughout the hospital via shedding of skin from different individuals. This could also reflect naturally large population variation present within the skin of a single individual, as has been reported for skin-associated bacteria [40, 41].

In the current analysis, most of the samples contained one dominant eukaryotic genotype, presumably one well adapted to the habitat, but other allele variants indicate the presence of lower-abundance genotypes (Fig. 4b). Given this dominance, it was possible to directly estimate genome heterozygosity. Prior studies have reported that *C. albicans* grows clonally in vivo [42], yet *Candida*, when expressing a certain phenotype, undergoes mating [42], most likely via a parasexual cycle [38]. For *C. albicans*, the measured heterozygosity was comparable to that of previously sequenced isolate genomes [34, 39]. Despite high heterozygosity of *C. albicans*, we see low strain heterogeneity. It has been hypothesized that *C. albicans* mating may occur primarily on the skin [43]. We speculate there may be more strain heterogeneity on the skin or other areas of the human microbiome besides in the gut, as it is probable that heterozygosity in *Candida* populations in the human and room microbiomes arises due to mating with distinct coexisting strains.

The heterozygosity measurements of all other fungi except *Malassezia* were low, possibly indicating diversity reduction due to inbreeding and/or strong selection for specific alleles. We speculate that this reflects a long history of colonization of a habitat type that strongly selects for a specific genotype, so genome structure reflects the relatively low probability of recombination with strains with divergent alleles (in other words, the presence of gut-adapted and sink-adapted strains). However, without the availability of similar genomes to compare to from other habitats, we cannot rule out genetic bottlenecks that took place prior to introduction to the hospital.

An important aspect of the current study is the sequencing of reagent controls, which allowed us to identify *P. lilacinum* as a likely contaminant. It is interesting to note that peak allele frequency analysis indicated high population microdiversity for the contaminant. Genomic microdiversity of the reagent-associated population may indicate its long-term persistence in the reagents, analogous to that shown for *Delftia* metagenome contamination that was present in Pippin size selection cassettes for many years [44]. Given the increasing use of metagenomic sequencing for pathogen detection and prior reports of *P. lilacinum* as both a contaminant and disease agent [45, 46], it will be important to rule out a reagent source of *P. lilacinum* in future diagnostic studies.

### Premature infants are colonized by eukaryotes early in life

Six percent of infants in this study were colonized by fungi, lower than most previous studies of infants [2, 12–14]. Compared to shotgun sequencing, DGGE and ITS methods should be more sensitive due to the use of PCR, and thus may be more suitable for broad ecological surveys. However, the ability to amplify very rare sequences from organisms present at exceedingly low abundance levels complicates interpretation of the measured colonization frequencies. Our shotgun sequencing-based methods provide a more balanced view of community composition than methods that rely on PCR, and detection of populations that comprise more than ~ 0.05% of the community DNA is possible with read-mapping (Additional file 1: Table S1; Additional file 6: Figure S2). Further, whole-community sequencing measures the relative abundance of eukaryotes in the context of the whole community, something that cannot be done using ITS, DGGE, or culturing-based methods. Fungi are generally considered low-abundance members of the gut microbiome [1], yet in this study, they reached levels as high as 55%, 78%, and 96% of the entire community **(**Fig. 2**)**. Differences in fungal communities during early life are known to have effects on infant health later in life [8], and it remains to be seen if extreme abundance levels like this have long-lasting effects.

All infants profiled in this study received 2–7 days of prophylactic antibiotics upon birth, meaning antibiotic use is highly correlated with earlier days of life (Additional file 7: Table S4). While both antibiotic administration and DOL were significantly correlated with eukaryote abundance, consistent with previous studies of fungal colonization of low birth weight infants [2, 47], infants who received antibiotics later in life were not colonized by eukaryotes. This suggests that day of life is the more important factor. However, eukaryotes may have not been detected in later collected microbiome samples from those infants due to increased relative abundance of bacteria. In other words, the sensitivity of the shotgun sequencing method may be insufficient to detect fungi that persist at low abundance.

Interestingly, permutation testing revealed that fungi colonized the same infants more often than expected by random chance. There may be several explanations for this phenomenon. For example, some infants may be more genetically predisposed to fungal colonization. Alternatively, fungi may interact synergistically, with the first colonizing species establishing a niche in the gut that makes it more suitable for other fungi. Should this effect prove to be important, it may help to explain how fungal colonization contributes to development of asthma or allergies [8].

### Differences in colonization patterns of NICU sinks and surfaces

Yeasts of the genus *Malassezia*, a common member of the skin microbiome [5, 30], dominated NICU surfaces.[5, 32]. This result is analogous to findings of previous studies, which showed that typically skin-associated bacteria dominate consortia associated with hospital surfaces and parts of other built environments [19, 26, 27, 48, 49].

The same eukaryotes were never detected in sinks and surfaces, and the sinks hosted a comparatively diverse and variable eukaryotic community (Fig. 3). Sinks are inherently heterogeneous environments with different moisture levels and chemical conditions. Punctuated cleaning events may also give rise to temporal variation. *Diptera* S2_005_002R2 (fly), which was present in only one sink sample, may be explained by sequencing of sink-associated eggs, as no macroscopic organisms were detected during the collection process. Recent studies have suggested that insects play significant roles in the dispersal of fungi, and this may occasionally occur in the NICU [50].

The other metazoan detected, the worm *Rhabditida* S2_005_001R2, was found in sinks from multiple rooms and samples collected months apart. These organisms may also be a source of fungi, and like the fly, could impact the overall NICU microbiome. Intriguingly, the partial genome appears to derive from an organism that is equally related to a bovine lungworm and *Caenorhabditis elegans* and is potentially novel at the class level (Fig. 1). Although we cannot evaluate its medical importance, the organism may have been macroscopically described but lack of a reference genome prevents identification.

## Conclusions

We applied genome-resolved metagenomics to study eukaryotes in the gut microbiomes of infants and their NICU rooms and detected eukaryotes associated with pathogenesis of immunocompromised humans, commensals of human skin, and fungi typical of environments such as soil and drain pipes. Genomic analysis of diploid organisms found low rates of heterozygosity that may be explained by persistence of hospital-associated lineages in environments that impose strong selective pressure. The application of this approach in other contexts should greatly expand what is known about eukaryotic genomic diversity and population variation.

## Methods

### Subject recruitment, sample collection, and metagenomic sequencing

This study made use of many different previously analyzed infant datasets. These datasets have previously published descriptions of the study design, patient selection, and sample collection, and are referred to as NIH1 [51, 52], NIH2 [19], NIH3 [53], NIH4 [54], Sloan2 [19], and SP_CRL [55]. Infants were chosen for inclusion in this study irrespective of fungal disease state. Negative extraction controls were performed and sequenced during the sequencing of the Sloan2 cohort. The last well of the extraction block (H12) was left empty, and this well was treated the same as all other samples throughout the extraction protocol. It is therefore a control for the kit reagents, the sterility of the kit tubes/plates, and the aseptic technique of the technician who performed the extraction. S2_CON_001E1, S2_CON_002E1, and S2_CON_003E1 were all on different extraction blocks, and S2_CON_002E2 was a second well on the same block as S2_CON_002E1.

This study also involved the collection and processing of an additional 269 samples from 53 infants. Newly collected infant fecal samples followed the same sample collection and DNA extraction protocol as described previously [53, 56]. Metagenomic sequencing of newly collected infant fecal samples was performed in collaboration with the Functional Genomics and Vincent J. Coates Genomics Sequencing Laboratories at the University of California, Berkeley. Library preparation on all samples was performed using the following basic protocol: (1) gDNA shearing to target a 500 bp average fragment size was performed with the Diagenode Bioruptor Pico, (2) end repair, A-tailing, and adapter ligation with an Illumina universal stub with Kapa Biosystems Hyper Plus Illumina library preparation reagents, and (3) a double AMpure XP bead cleanup, followed by indexing PCR with dual-matched 8 bp Illumina compatible primers. Final sequence ready libraries were visualized and quantified on the Advanced Analytical Fragment Analyzer, pooled into 11 subpools based on mass, and checked for pooling accuracy by sequencing on Illumina MiSeq Nano sequencing runs. Libraries were then further purified using 1.5% Pippin Prep gel size selection assays collecting library pools from 500 to 700 bp. Pippin pools were visualized on fragment analyzer and quanted with Kapa Illumina library quant qPCR reagents and loaded at 3 nM. The 11 pools were then sequenced on individual Illumina HiSeq4000 150 paired-end sequencing lanes with 2% PhiX v3 spike-in controls. Post-sequencing bcl files were converted to demultiplexed fastq files per the original sample count with Illumina’s bcl2fastq v2.19 software. New metagenomic data was processed in the same manner as in the prior studies, and as described previously [54].

Environmental metagenomes were described and published previously as part of the Sloan2 cohort study [19]. All samples were collected over a roughly one-year period from the same NICU at the University of Pittsburgh Magee-Womens Hospital. In order to generate enough DNA for metagenomic sequencing, DNA was collected from multiple sites in the NICU and combined into three separate pools for sequencing. Highly-touched surfaces included samples originating from the isolette handrail, isolette knobs, nurses hands, in-room phone, chair armrest, computer mouse, computer monitor, and computer keyboard. Sink samples included samples from the bottom of the sink basin and drain. Counters and floors consisted of the room floor and surface of the isolette. See previous publications for details [19, 57].

### Eukaryotic genome binning and gene prediction

Reads from each sample were assembled independently using IDBA-UD [58] under default settings. A co-assembly was also performed for each infant, consisting of reads from all samples taken from that infant concatenated together. Binning assembled sequence scaffold into eukaryotic genomes was performed using a EukRep-based pipeline, described in detail in West et al. [30]. In cases where time-series data were available, samples were pre-binned using time-series information and eukaryotic bins were then subsequently identified with EukRep. In cases where multiple genomes of the same organism were recovered from multiple samples from the same infant, the most complete genome was selected for further analysis. In addition to the gene prediction methodology outlined previously [30], a second homology-based gene prediction step was performed. Ribosomal S3 (rpS3) proteins were identified in genomes using a custom ribosomal protein S3 (rpS3) profile HMM, and identified sequences were searched against the NCBI database [59] and UniProt [60] using BLAST [61]. For each de novo-assembled genome, gene sets for the top 1–3 most similar organisms were used as homology evidence for a second-pass gene prediction step with AUGUSTUS [62], as implemented in MAKER [63]. For *Rhabditida* S2_005_001R2, first-pass gene predictions were used, as homology evidence decreased overall estimated genome completeness. Genome completeness was estimated using BUSCO [64] and is based on the number of detected single-copy orthologs. N50 was calculated using the program checkM [65].

To verify bins, the taxonomy of each scaffold was determined by searching gene sequences against the UniProt database [53]. All bins were found to have a consistent phylogenetic signal, except the bin created from sample S2_009_000R2. Scaffolds had similar GC content and sequencing coverage, but were either dominated by genes with homology to the class *Sordariomycetes* or *Eurotiomycetes*. Scaffolds from the original “megabin” were split into two separate bins based on this phylogenetic signal, resulting in the genomes *Nectria haematococca* S2_009_000R2 and *Exophiala* sp. S2_009_000R2. Gene prediction was run again for both of these genomes, as described above.

### Phylogenetic analyses

In order to construct a phylogenetic tree, *rpS3* proteins from each de novo genome were detected as described above and searched against the NCBI database using BLAST. Protein sets of the 3–5 most similar organisms on NCBI were downloaded for inclusion. Other phylogenetically important genomes, such as *A. thaliana*, were included as well. For each protein set, 16 ribosomal proteins (bacterial ribosomal protein names L2, L3, L4, L5, L6, L14, L15, L16, L18, L22, L24, S3, S8, S10, S17, and S19) were identified using custom-built hidden Markov models (HMMs) with HMMER [66], using the noise cutoff (NC). The 16 ribosomal protein datasets were then aligned with MUSCLE [67] and trimmed by removing columns containing 90% or greater gaps. The alignments were then concatenated. A maximum likelihood tree was constructed using RAxML v.8.2.10 [68] on the CIPRES web server [69] with the LG plus gamma model of evolution (PROTGAMMALG) and with the number of bootstraps automatically determined with the MRE-based bootstrapping criterion. The constructed tree was visualized with Interactive Tree of Life (ITOL) [70].

Average nucleotide identity (ANI) between binned genomes and reference genomes was determined with dRep [35]. Resulting whole genome ANI values were used in combination with a 16 ribosomal protein phylogenetic tree to determine the taxonomy of de novo genomes. For genomes without a species-level taxonomy, genomes were searched against the entire NCBI nucleotide database using BLAST. This resulted in a species-level call for *Malassezia restricta* S2_018_000R1*.* For genomes without a genus-level taxonomy (*Rhabditida* S2_005_001R2 and *Diptera* S2_005_002R2), an additional step was taken. Mitochondrial *COI* genes were identified by searching *D. melanogaster* and *C. elegans COI* genes against our PRODIGAL [71] predicted genes sets with UBLAST [72]. Significant hits from our protein sets were then searched against the Barcode Of Life Database (BOLD) [31] and NCBI in order to identify sequences with high identity to our novel genomes. No significant hits were identified.

### Mapping-based genome detection

To detect eukaryotes in an assembly-free manner, reads were mapped to a curated genome collection. This genome collection consists of all fungal genomes in RefSeq (accessed 9/14/17) [73], as well as genomes assembled in this study with no close representatives in RefSeq (average nucleotide identity of 90% or higher according to Mash [74]). The six genomes with no close representatives in RefSeq were *Malassezia restricta* S2_018_000R1, *Diptera* S2_005_002R2, *Exophiala* sp. S2_009_000R2, *Verruconis* sp. S2_005_001R2, and *Rhabditida* S2_005_001R2. *Candida parapsilosis* CDC317 was also included, as there were no genomes of *C. parapsilosis* in RefSeq.

Reads from all samples were mapped to this reference genome list using Bowtie 2 [75]. To determine which organisms were present in each sample, we primarily relied on breadth of coverage as reported by strainProfiler (https://github.com/MrOlm/strainProfiler). In NICU samples, all genomes with 50% breadth of coverage or above were considered present. For infant samples, reads resulting from concatenating all samples belonging to the same infant were first used to determine which fungi are reliably detected. Genomes with 50% breadth of coverage or above were considered present with two exceptions, *Malassezia pachydermatis* and *Malassezia sympodialis*, at ~ 0.2 and 0.4 breadth, respectively. Considering the extensive and distributed breadth of coverage for these genomes (Additional file 3: Figure S1C), they were considered present in the infant despite having low breadth of coverage overall. Reads from each individual sample from each infant were then mapped to all fungi considered to be present in that infant to determine changes over time. Relative abundance of genomes was determined using the formula: (number of reads mapping to genome/total number of reads in sample).

The lowest coverage genome with this breadth threshold was 1.1× coverage. To determine the limit of detection, we first determined the relative abundance needed to achieve 1.1× coverage using the median infant co-assembly depth (27.5 Gb) and the median eukaryotic genome length in our database of organisms that were detected at least once (13.7 Mbp). We then calculated the limit of detection using the formula ((min coverage × median length)/median co-assembly depth). This lead to an estimated limit of detection of 0.05% relative abundance for infant fungi detection, through this number has significant variability depending on how deep each individual infant was sequenced.

### Negative extraction control analysis

Sequences resulting from negative extraction controls were computationally processed in an identical manner to other samples. Reads from all control samples were mapped to the curated genome collection described above, and the relative abundance of all genomes with at least 10% breadth was plotted in Additional file 3: Figure S1. The program strainProfiler (https://github.com/MrOlm/strainProfiler) was used to compare reads in sample S2_CON_000E3 to *P. lilacium* genomes assembled in this study and all publically available *P. lilacinum* genomes. Version 0.2 of the program was run with default settings, resulting in an average nucleotide identity measure between sample S2_CON_000E3 and all *P. lilacinum* genomes. Next, dRep v1.4.3 [35] was used to compare the *P. lilacinum* genomes with each other using the command “dRep cluster --SkipMash”. The resulting distance matrix was merged with the values generated from strainProfiler to generate the dendrogram in Additional file 3: Figure S1B. Full code for implementation is available at https://github.com/MrOlm/InfantEukaryotes.

All publically available *Malassezia* genomes were acquired by searching for the term “Malassezia” in the assembly section of NCBI and downloading them manually. Genomes were compared to each other, and representative genomes were chosen using dRep v1.4.3 and the commands “dRep compare --SkipMash” and “dRep choose --noQualityFiltering -sizeW 0.5”. A concatenation of all negative extraction control sequences was then mapped to the resulting genomes using Bowtie 2. Custom scripts were used to determine the breadth of coverage of each 10,000 bp window of each fungal genome in each sample, and each window with at least 50% breadth was marked with a tick using Circos [76] to visualize. Open source code detailing this analysis is available at https://github.com/MrOlm/InfantEukaryotes.

To determine the expected breadth of coverage (percentage of genome base pairs with at least one read) for a given depth of coverage (average number of reads at any given genome base pair), a simulation was performed. Metagenomic reads were first simulated for *Escherichia coli* and *Candida albicans* reference genomes using pIRS (https://github.com/galaxy001/pirs). Simulated reads were mapped back to the original reference genome, and the resulting .bam file was subset 20 times to simulate various depths of coverage. The breadth and depth of coverage was plotted and an exponential line of best fit was calculated using SciPy [77]. The line had an *R*^2^ value over 0.99 and was defined using the equation: breadth = (− 1 ×*e*^(− 0.883 × coverage)) + 1. This equation was used to determine the expected breadth of coverage for a given depth of coverage.

### Statistical analyses and generation of MDS plot

To compare the eukaryotic communities present in NICU room samples, multidimensional scaling (MDS) based on Bray-Curtis distance was performed. The Bray-Curtis distance was calculated based on the relative abundance of each eukaryote present in a sample using the python library SciPy (command scipy.spatial.distance.braycurtis) [77]. Eukaryotes with at least 50% breadth of coverage were considered present in a sample. MDS was performed on the resulting all-vs-all distance matrix using the python library sklearn (command sklearn.manifold. MDS) [78]. MDS was plotted using a custom function in Matplotlib [79]. Stress was calculated using sklearn. Open source code detailing this analysis is available at https://github.com/MrOlm/InfantEukaryotes.

We tested for significant associations between samples containing eukaryotes and various forms of metadata using the python SciPy package [77]. Included were six pieces of continuous metadata (DOL, infant birth weight, etc.), 23 pieces of categorical metadata (specific antibiotics given and specific NICU room locations), and the phyla-level abundance of all bacterial genomes (seven total phyla) (Additional file 7: Table S4). Bacterial phyla-level abundance was determined by summing the relative abundance of all bacterial genomes present in a sample. Bacterial genomes for previously sequenced samples are available in a previous publication [54], and bacterial genomes for newly sequenced genomes were binned using the same methods. Metadata was filtered such that between 20 and 80% of values were non-zero in both samples containing eukaryotes and samples not containing eukaryotes. This resulted in a total of 13 pieces of metadata for statistical testing (Additional file 7: Table S4).

In order to eliminate statistical bias introduced through sampling the same infant multiple times, one sample from each infant was chosen for statistical tests. If the infant was not colonized by a eukaryote, the sample was chosen at random. If the infant was colonized by a eukaryote, the sample with the highest eukaryotic abundance was chosen. Samples were considered to have a eukaryote present if the sum of the relative abundance of eukaryotes with at least 50% breadth was at least 0.1% relative abundance. Fisher’s exact test was used for categorical metadata, and Wilcoxon rank-sum test was used for continuous data. Benjamini-Hochberg *p* value correction [80] was performed to account of multiple hypothesis testing. The results of all statistical tests are provided in Additional file 8: Table S5. Open source code detailing this statistical analysis is available at https://github.com/MrOlm/InfantEukaryotes.

A permutation test was performed to determine if fungi were distributed randomly among the infants. First, 100,000 trials were run where each trial consisted of randomly selecting 13 individuals with replacement from a total population of 161 individuals. The number of infants chosen was determined for each trial, and an empirical *p* value was determined based on how many trials had 10 of less infants chosen. Open source code detailing this statistical analysis is available at https://github.com/MrOlm/InfantEukaryotes.

### Ploidy, heterozygosity, and population microdiversity

In order to identify variants, reads from the sample in which a particular genome was binned from were mapped back to the de novo assembled genome using Bowtie 2 [75] with default parameters. The PicardTool (http://broadinstitute.github.io/picard/) functions “SortSam” and “MarkDuplicates” were used to sort the resulting sam file and remove duplicate reads. FreeBayes [81] was used to perform variant calling with the options “--pooled-continuous -F 0.01 -C 1.” Variants were filtered downstream to include only those with support of at least 10% of total mapped reads in order to avoid false positives. Furthermore, to avoid including variants as a result of mismapping reads, variants were filtered to include only those with coverage depth within a range of the average genome coverage plus or minus half of the genome mean coverage. SNP read counts were calculated using the “AO” and “RO” fields in the FreeBayes vcf output file. Multiallelic sites were defined as sites with two or more non-reference alleles. Variants were called using the same methodology for both simulated read datasets and isolate genomes. Variants were used to determine ploidy, heterozygosity, and population microdiversity as described in the “Results” section. Source code with full implementation details is available at https://github.com/MrOlm/InfantEukaryotes.

To confirm that multiallelic sites are not the result of non-specifically mapped reads from the bacterial community, we fragmented with pIRS (https://github.com/galaxy001/pirs) a diploid *C. parapsilosis* genome into simulated reads and added these reads to an infant gut metagenome sample without *C. parapsilosis*. The resulting read dataset along with a separate dataset comprised of only the simulated reads were then mapped to the original *C. parapsilosis* genome. No additional variants were detected between the sample with metagenomic reads and the sample without, indicating non-specifically mapped reads from bacterial community members have a minimal effect.

In order to determine the effect of stochastic read coverage on variant frequencies, simulated haploid, diploid, and triploid genomes were generated using the pIRS (https://github.com/galaxy001/pirs) diploid command with the *C. albicans* P57072 reference genome. The command was used once to generate a diploid genome and twice to generate a triploid genome. Simulated reads were then generated for each genome using the pIRS simulate command at 10×, 50×, and 100× coverage. Assemblies and raw reads were downloaded for both *C. albicans* A48 and *C. parapsilosis* CDC317 from NCBI to be used as example isolate genomes for comparison. Based on this analysis, only the two genomes with at least 50× coverage were included in peak allele frequency analysis.

Genome aneuploidy was analyzed in two ways. First, reads from each sample were mapped back to genomes assembled from that sample. The coverage of each scaffold was determined in 10 kbp windows, and the coverage of all windows for each scaffold over 10 kbp was plotted. Plots were then analyzed for scaffolds with differing coverage, indicative of the presence of multiple copies of a subset of the chromosomes **(**Additional file 13: Figure S6**)**. Second, reads from samples with genomes assembled from them were mapped to the closest available reference genome. The same procedure was then performed with these reference genomes in all cases where at least 80% of the genome was covered by reads. This allowed the determination of aneuploidy on the whole-chromosome level (Additional file 14: Figure S7). Both methods agreed that in all cases, no aneuploidy was detected.

## Additional files

Additional file 1: **Table S1.** Sequencing metadata for all infant and room metagenomic samples. (CSV 69 kb)

Additional file 2: **Table S2.** Detailed information about genome assemblies.(CSV 1 kb)

Additional file 3: **Figure S1.** Fungal contaminants are present in negative extraction controls. **(A)** Relative abundance of eukaryotes in four sequenced extraction controls (based on read mapping). **(B)** P. lilacinum sequences from the extraction control (red) closely resemble sequences recovered from gut and room samples (blue), and are distinct from publically available genomes (black). **(C, D)** Each ring shows the breadth of coverage across **(C)** four different Malassezia genomes or **(D)** a Purpureocillium lilacinum reference genomes for an individual sample. Red, blue, and green rings are extraction controls, NICU room samples, and premature infant guts samples respectively. Each colored tick represents a 10 kb window in which the breadth of coverage is at least 50%. (PNG 461 kb)

Additional file 4: **Figure S9.** Breadth of coverage vs. depth of coverage. The breadth of coverage and depth of coverage resulting from mapping simulated reads of different depths back to the reference genome. The equation for the line of best fit and R2 value are also shown. (PNG 16 kb)

Additional file 5: **Table S3.** Mapping-based abundance of eukaryote genomes in all samples. (CSV 24934 kb)

Additional file 6: **Figure S2.** The sequencing depth and relative abundance needed to detect eukaryotic genomes of various lengths at 1x coverage. (PDF 112 kb)

Additional file 7: **Table S4.** Metadata for statistical associations. (CSV 349 kb)

Additional file 8: **Table S5.** Statistical associations of samples containing eukaryotes with metadata. (CSV 1 kb)

Additional file 9: **Figure S8.** Metagenomic contaminants display similar relative abundance patterns to genuine community members. The scaled relative abundance of each eukaryote colonizing an infant is shown. Numbers on the right indicate the maximum relative abundance of the organism in that infant, and grey dividing lines indicate 0% relative abundance. Dots on the line-plots indicate days of life on which fecal samples were collected and sequenced. Both genuine community members and metagenomic contaminants display a pattern of decreasing relative abundance as infants age, suggesting that the decrease may be due to bacterial grown rather than fungal decline. (PNG 100 kb)

Additional file 10: **Figure S3.** *C. parapsilosis* genomes from the NICU sink of infant S2_005 and gut of infant N3_182 were more similar to reference genomes than each other. (PNG 138 kb)

Additional file 11: **Figure S4.** Effect of coverage on variant frequency determination as assessed through simulation of metagenomic reads. (PNG 144 kb)

Additional file 12: **Figure S5.** Raw variant frequency graphs used to determine ploidy of all de novo assembled genomes. (PDF 448 kb)

Additional file 13: **Figure S6.** Determination of aneuploidy for all de novo assembled genomes based on scaffold coverage. The coverage of each 10kb window of each scaffold is shown. Scaffolds are ordered from largest to smallest, and rotate between red and black colors. No large portions of chromosomes were detected as having a multiple of 1/2x the coverage of the genome average as would be expected from a diploid genome. (PNG 2848 kb)

Additional file 14: **Figure S7.** Alternative mapping-based determination of aneuploidy for genomes with high quality reference genomes. No large portions of chromosomes were detected as having a multiple of 1/2x the coverage of the genome average as would be expected from a diploid genome. (PNG 1497 kb)

Additional file 15: **Figure S10.** Population heterogeneity of the P. lilacinum metagenomic contaminant. Histogram of the frequencies of the four most abundant variants at each variant site in the genome. Black, red, dark blue and light blue bars indicate the abundances of the most abundant, second, third and fourth most abundant variant, respectively. (PNG 19 kb)
