# Supplementary figures and images for: Genome-resolved metagenomics of eukaryotic populations during early colonization of premature infants and in hospital rooms
Source: Microbiome. 2019 Feb 15;7:26. doi: 10.1186/s40168-019-0638-1 (PMC6377789; doi:10.1186/s40168-019-0638-1)

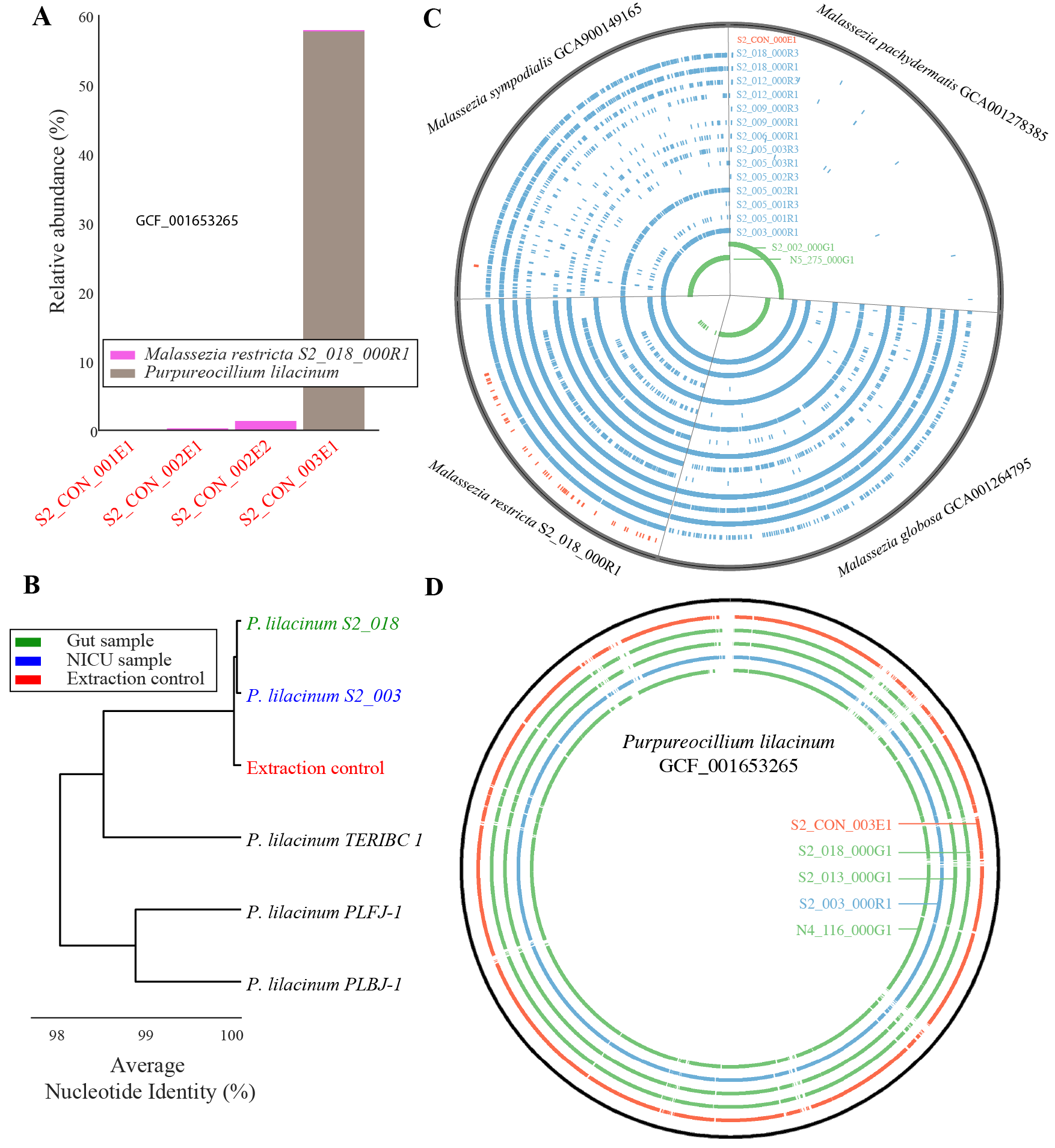

Supplement: Supplementary file 3 — Figure S1. Fungal contaminants are present in negative extraction controls. (A) Relative abundance of eukaryotes in four sequenced extraction controls (based on read mapping). (B) P. lilacinum sequences from the extraction control (red) closely resemble sequences recovered from gut and room samples (blue), and are distinct from publically available genomes (black). (C, D) Each ring shows the breadth of coverage across (C) four different Malassezia genomes or (D) a Purpureocillium lilacinum reference genomes for an individual sample. Red, blue, and green rings are extraction controls, NICU room samples, and premature infant guts samples respectively. Each colored tick represents a 10 kb window in which the breadth of coverage is at least 50%. (PNG 461 kb) [file 40168_2019_638_MOESM3_ESM.png]

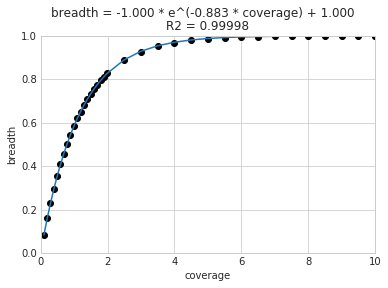

Supplement: Supplementary file 4 — Figure S9. Breadth of coverage vs. depth of coverage. The breadth of coverage and depth of coverage resulting from mapping simulated reads of different depths back to the reference genome. The equation for the line of best fit and R2 value are also shown. (PNG 16 kb) [file 40168_2019_638_MOESM4_ESM.png]

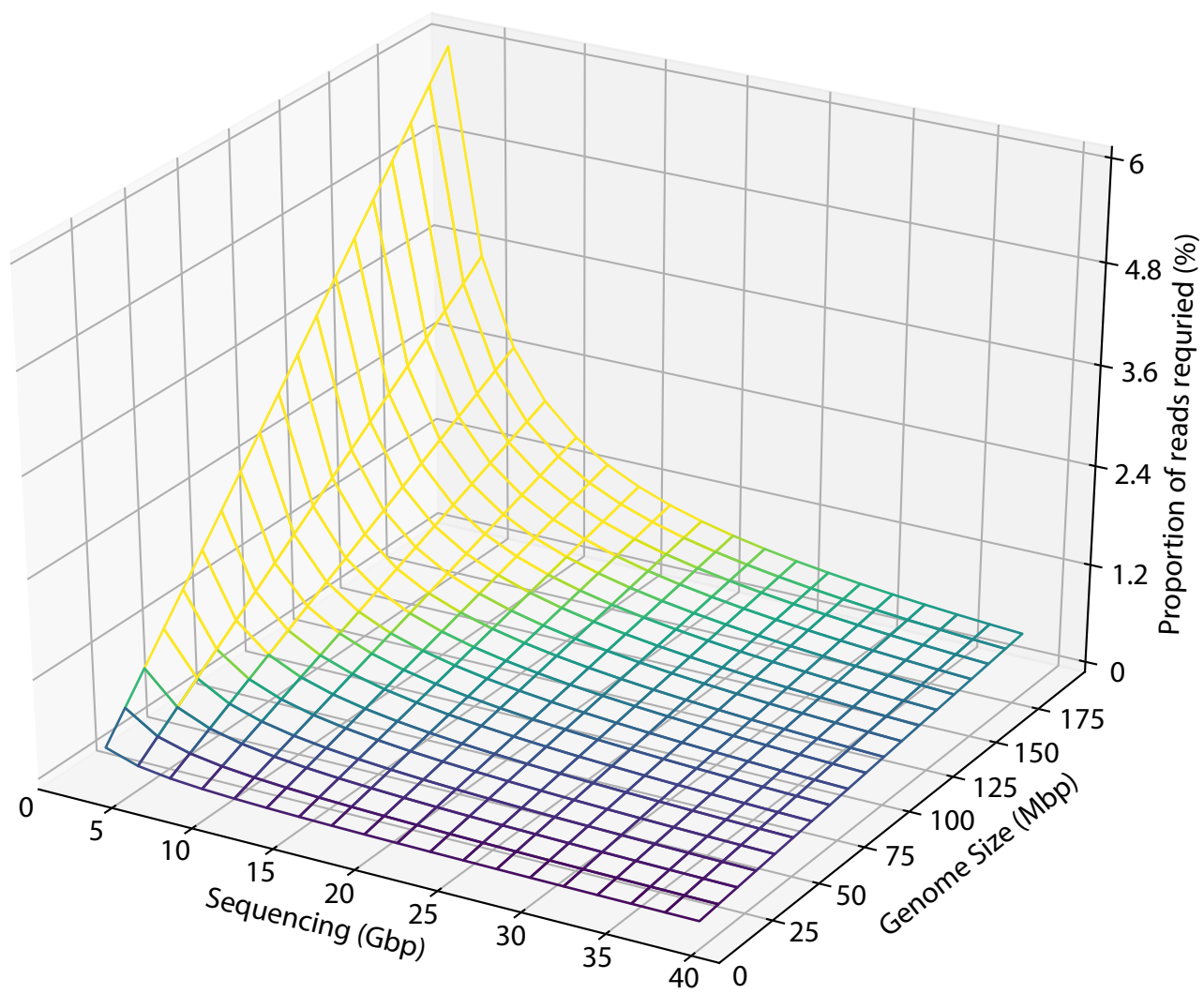

Supplement: Supplementary file 6 — Figure S2. The sequencing depth and relative abundance needed to detect eukaryotic genomes of various lengths at 1x coverage. (PDF 112 kb) [file 40168_2019_638_MOESM6_ESM.pdf]

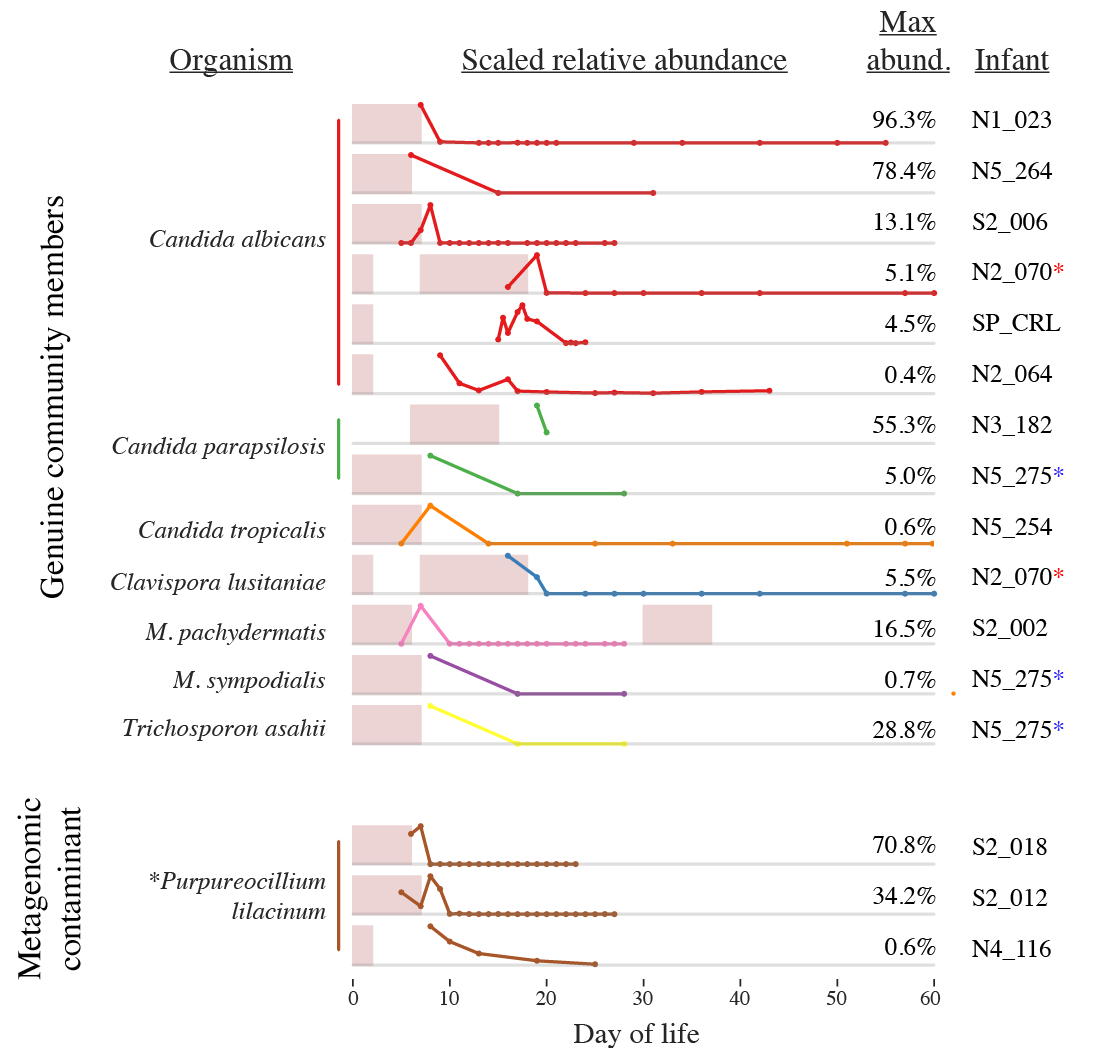

Supplement: Supplementary file 9 — Figure S8. Metagenomic contaminants display similar relative abundance patterns to genuine community members. The scaled relative abundance of each eukaryote colonizing an infant is shown. Numbers on the right indicate the maximum relative abundance of the organism in that infant, and grey dividing lines indicate 0% relative abundance. Dots on the line-plots indicate days of life on which fecal samples were collected and sequenced. Both genuine community members and metagenomic contaminants display a pattern of decreasing relative abundance as infants age, suggesting that the decrease may be due to bacterial grown rather than fungal decline. (PNG 100 kb) [file 40168_2019_638_MOESM9_ESM.png]

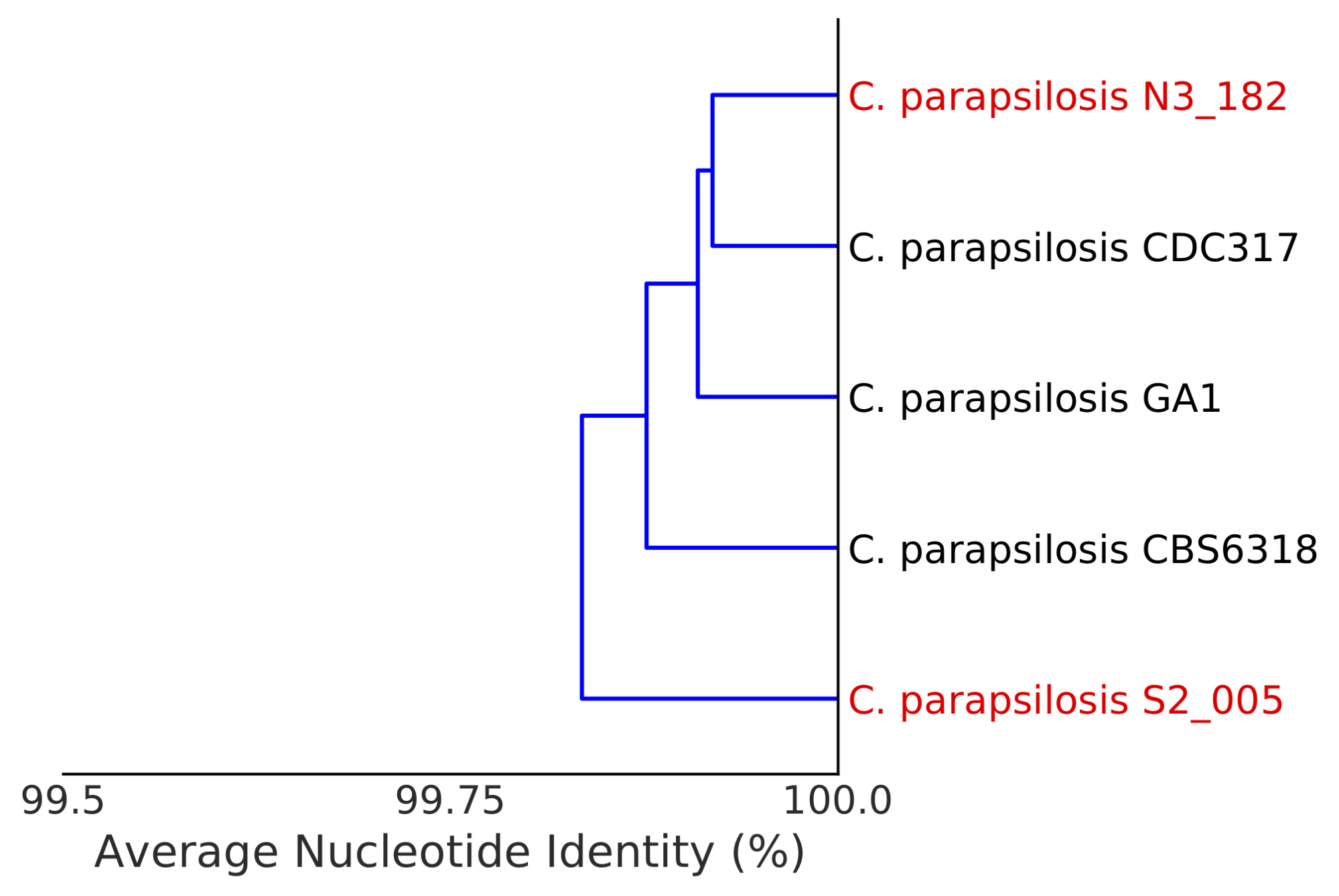

Supplement: Supplementary file 10 — Figure S3. C. parapsilosis genomes from the NICU sink of infant S2_005 and gut of infant N3_182 were more similar to reference genomes than each other. (PNG 138 kb) [file 40168_2019_638_MOESM10_ESM.png]

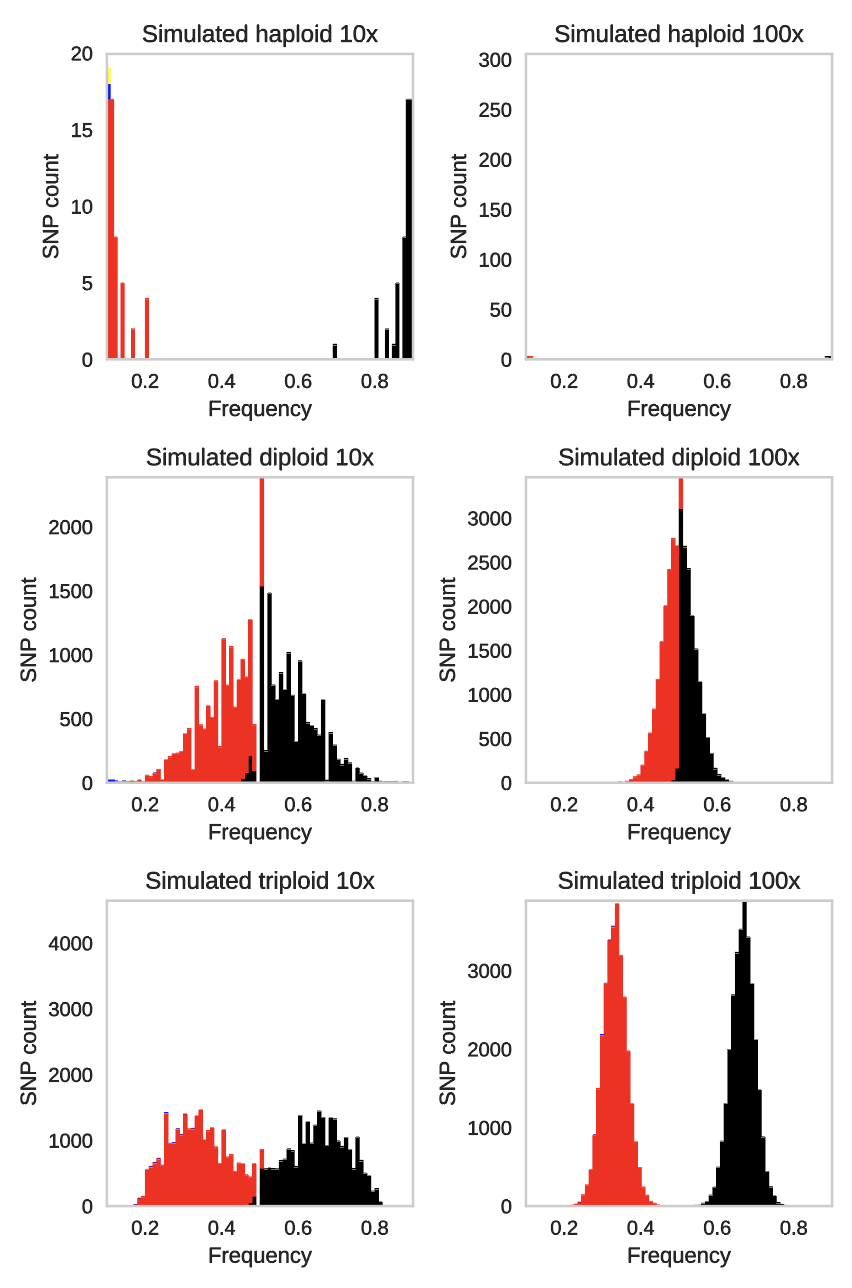

Supplement: Supplementary file 11 — Figure S4. Effect of coverage on variant frequency determination as assessed through simulation of metagenomic reads. (PNG 144 kb) [file 40168_2019_638_MOESM11_ESM.png]

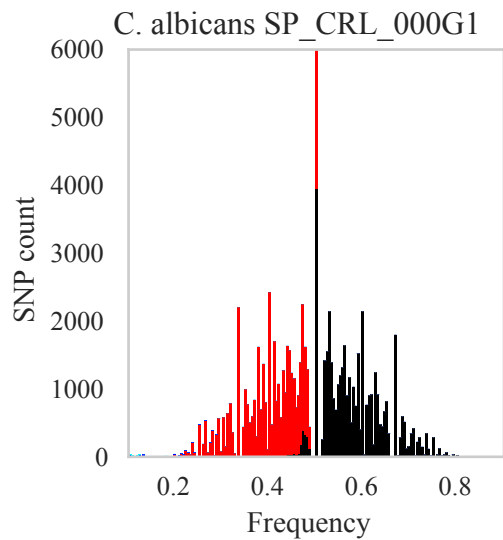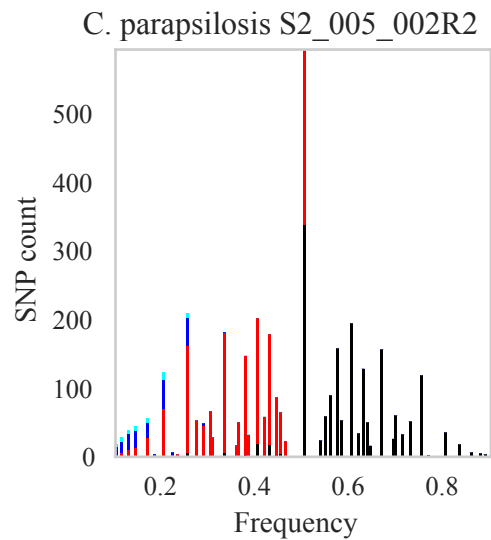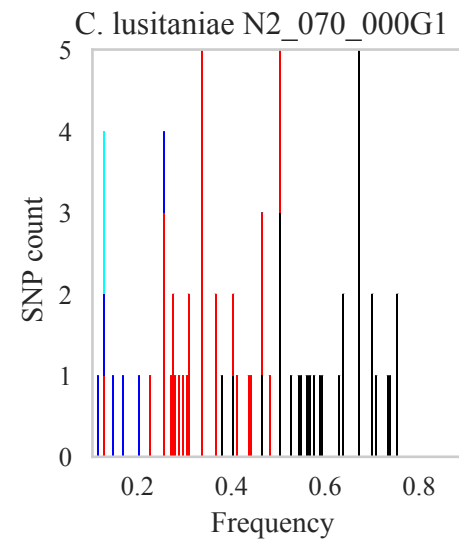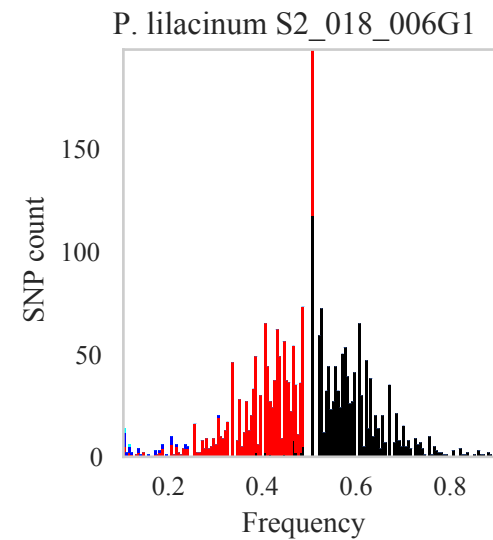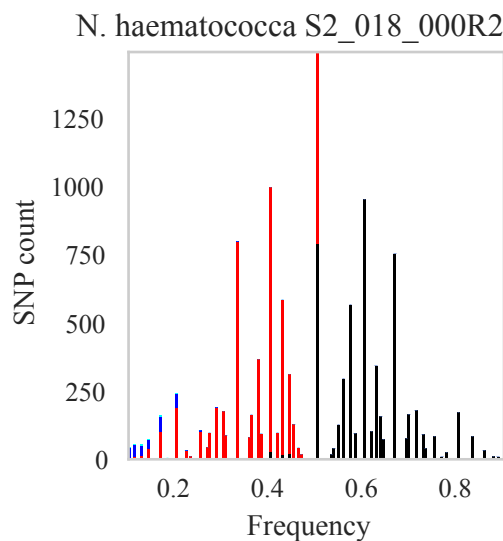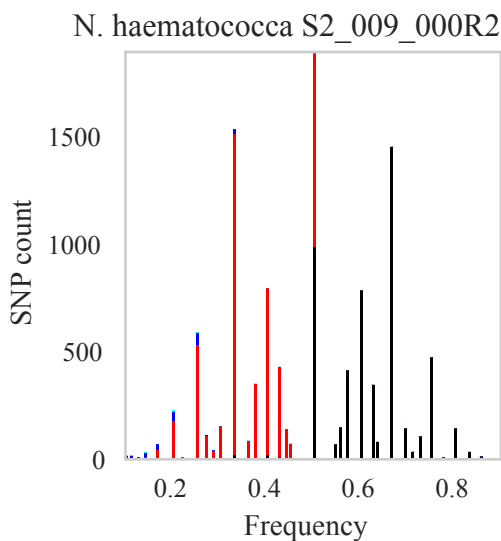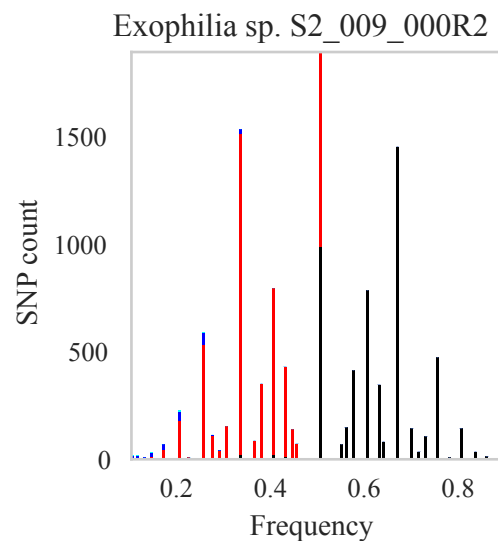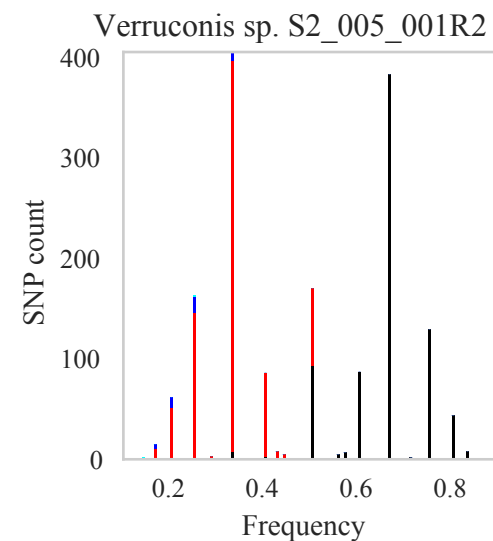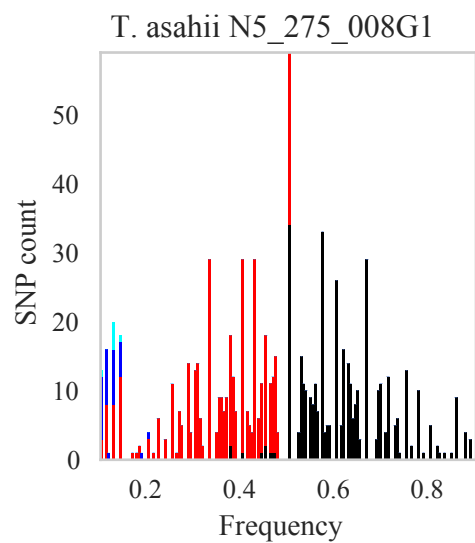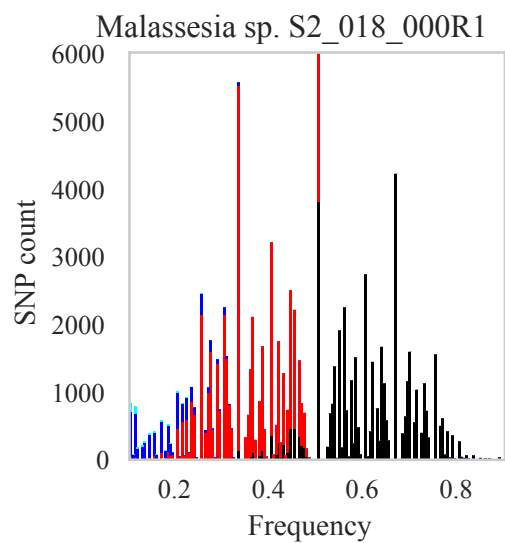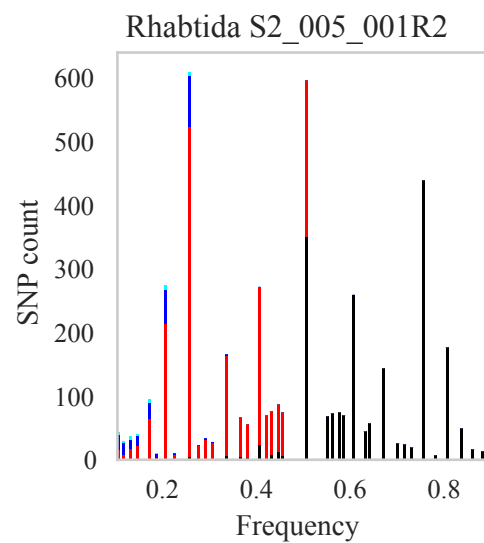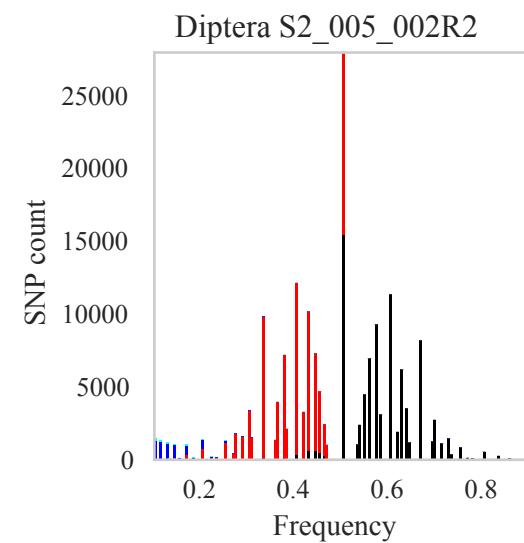

Supplement: Supplementary file 12 — Figure S5. Raw variant frequency graphs used to determine ploidy of all de novo assembled genomes. (PDF 448 kb) [file 40168_2019_638_MOESM12_ESM.pdf]

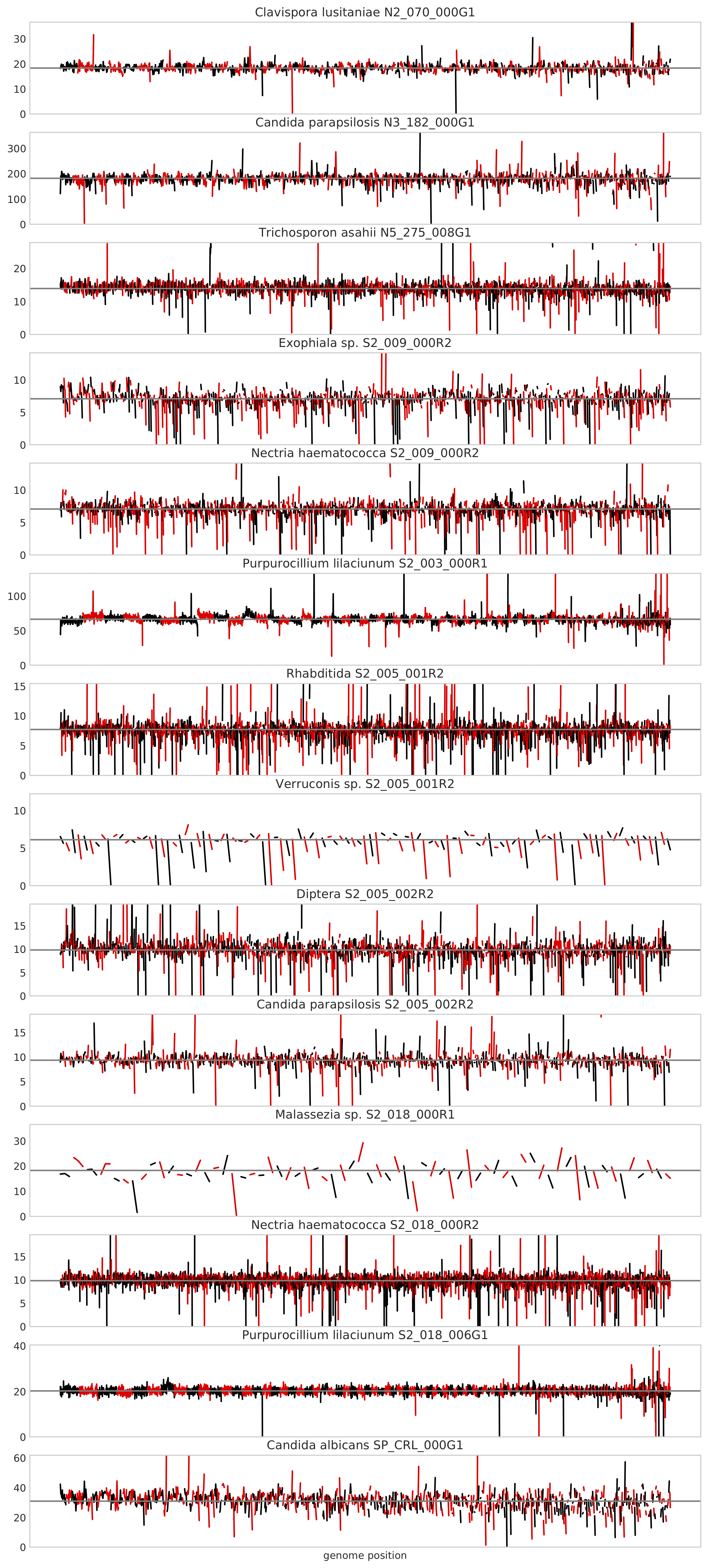

Supplement: Supplementary file 13 — Figure S6. Determination of aneuploidy for all de novo assembled genomes based on scaffold coverage. The coverage of each 10kb window of each scaffold is shown. Scaffolds are ordered from largest to smallest, and rotate between red and black colors. No large portions of chromosomes were detected as having a multiple of 1/2x the coverage of the genome average as would be expected from a diploid genome. (PNG 2848 kb) [file 40168_2019_638_MOESM13_ESM.png]

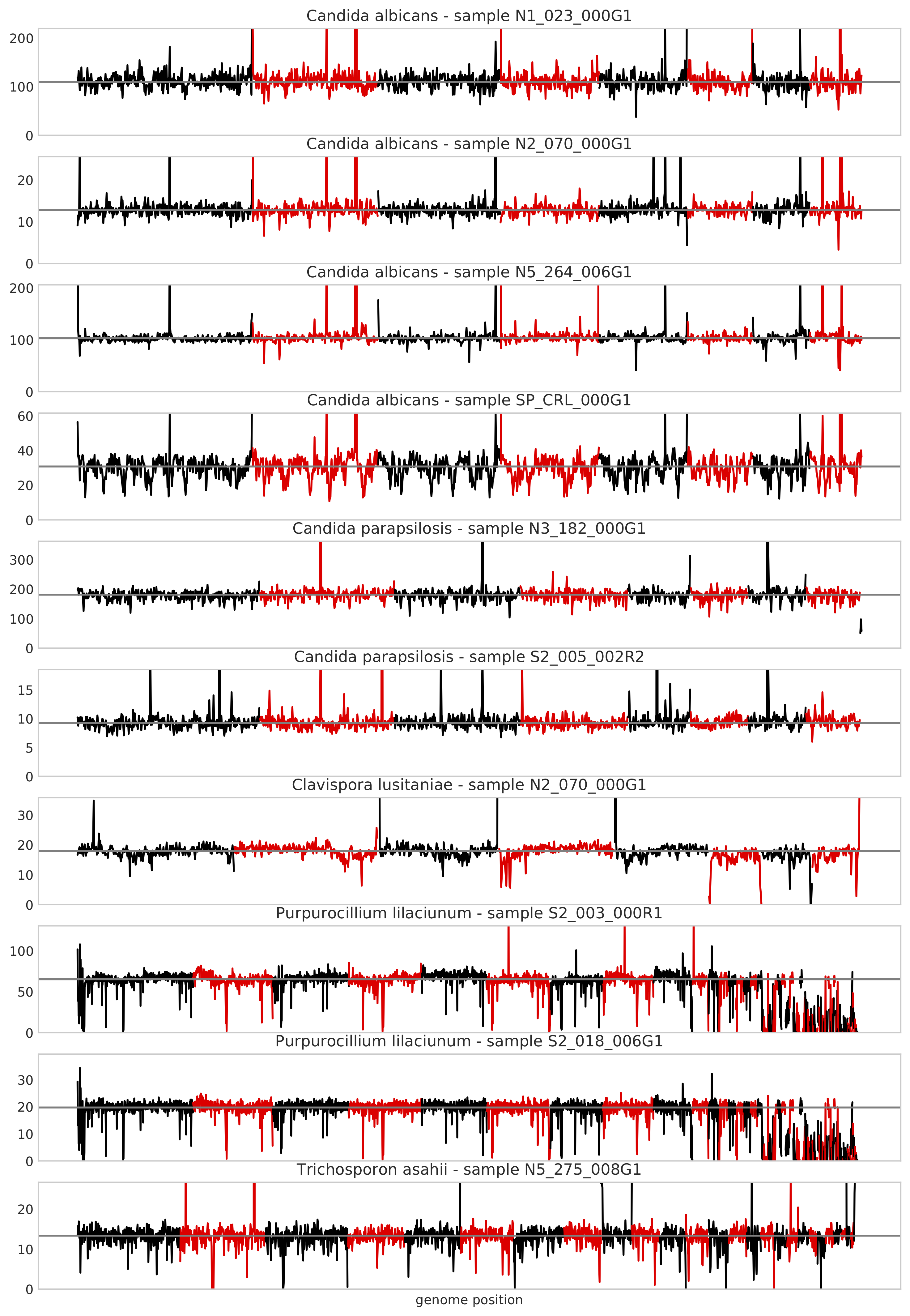

Supplement: Supplementary file 14 — Figure S7. Alternative mapping-based determination of aneuploidy for genomes with high quality reference genomes. No large portions of chromosomes were detected as having a multiple of 1/2x the coverage of the genome average as would be expected from a diploid genome. (PNG 1497 kb) [file 40168_2019_638_MOESM14_ESM.png]

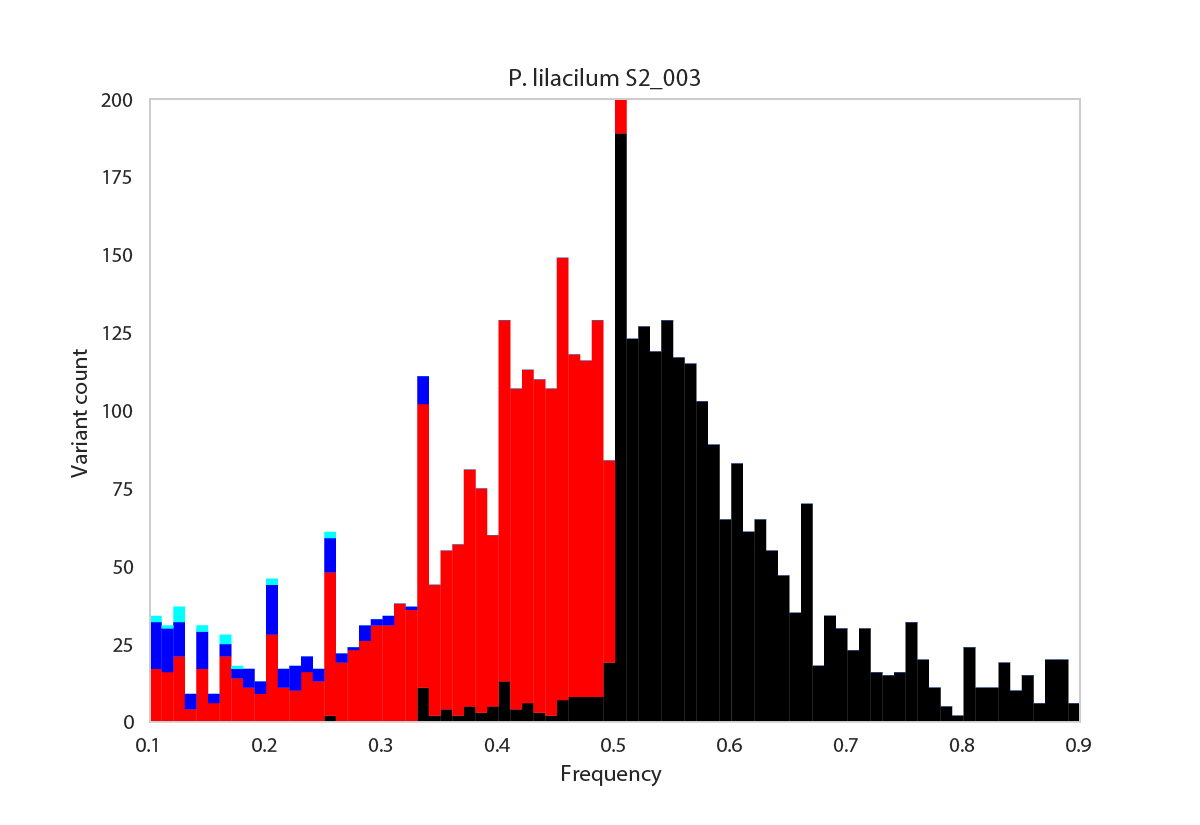

Supplement: Supplementary file 15 — Figure S10. Population heterogeneity of the P. lilacinum metagenomic contaminant. Histogram of the frequencies of the four most abundant variants at each variant site in the genome. Black, red, dark blue and light blue bars indicate the abundances of the most abundant, second, third and fourth most abundant variant, respectively. (PNG 19 kb) [file 40168_2019_638_MOESM15_ESM.png]
